# From the divergence of senescent cell fates to mechanisms and selectivity of senolytic drugs

**DOI:** 10.1098/rsob.220171

**Published:** 2022-09-21

**Authors:** Valentin L'Hôte, Carl Mann, Jean-Yves Thuret

**Affiliations:** CEA, CNRS, Institute for Integrative Biology of the Cell (I2BC), Université Paris-Saclay, Gif-sur-Yvette cedex, France

**Keywords:** cellular senescence, senolytics, mechanisms of action, cell survival, drug discovery, ageing

## Abstract

Senescence is a cellular stress response that involves prolonged cell survival, a quasi-irreversible proliferative arrest and a modification of the transcriptome that sometimes includes inflammatory gene expression. Senescent cells are resistant to apoptosis, and if not eliminated by the immune system they may accumulate and lead to chronic inflammation and tissue dysfunction. Senolytics are drugs that selectively induce cell death in senescent cells, but not in proliferative or quiescent cells, and they have proved a viable therapeutic approach in multiple mouse models of pathologies in which senescence is implicated. As the catalogue of senolytic compounds is expanding, novel survival strategies of senescent cells are uncovered, and variations in sensitivity to senolysis between different types of senescent cells emerge. We propose herein a mechanistic classification of senolytic drugs, based on the level at which they target senescent cells: directly disrupting BH3 protein networks that are reorganized upon senescence induction; downregulating survival-associated pathways essential to senescent cells; or modulating homeostatic processes whose regulation is challenged in senescence. With this approach, we highlight the important diversity of senescent cells in terms of physiology and pathways of apoptosis suppression, and we describe possible avenues for the development of more selective senolytics.

## Graphical abstract

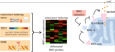

## 1. Introduction

Accumulating senescent cells are being found to drive a large number of pathologies. In the recent years, the therapeutic potential of senescent cell elimination has been demonstrated in mouse models of diseases and disorders as diverse as pulmonary fibrosis [1–3], type 1 and 2 diabetes [4–6], neurodegeneration [7–10] and atherosclerosis [11–13], among others. Pharmacological clearance of senescent cells is achieved with drugs called senolytics, which exhibit a significant toxicity towards senescent cells, with lesser effects on their proliferative or quiescent counterparts. Senolytics have been the object of intense research effort in the last 5–10 years—the first demonstration of selective pharmacological elimination of senescent cells dating back to 2013 [14], with the term senolytic being coined a few years later [15,16]. The fast-moving senolytic research landscape considerably evolved in the last 5 years: many clinical trials are now underway, innovative pharmacological strategies are being explored and the number of referenced senolytic drugs currently stands at above a dozen (table 1), and is likely to increase. It is now time to take a step back and comprehensively review and conceptualize senolysis.

**Table 1.** Notable senolytics: mechanisms and preclinical models.

senolytic compounds	notable members	class	mechanism	notable preclinical pathological models	references
BH3 mimetics	navitoclax, ABT-737, venetoclax, A1331852, A1155463	1	BH1–4 anti-apoptotic factors inhibition	diabetes, lung fibrosis, neurodegeneration, atherosclerosis, Covid-19, chemotherapy	[2–4,6,7,11,12,17–20]
MDM2 and USP7 inhibitors	P5091, UBX0101, nutlin-3a	2	p53 levels upregulation	chemotherapy, osteoarthritis, macular degeneration	[21–24]
dasatinib + quercetin	—	2	ephrin, AKT, PAI-2 inhibition	lung fibrosis, neurodegeneration, diabetes, ageing, Covid-19	[1,5,6,8–10,20,25,26]
FOXO4-p53 disrupters	FOXO4-DRI, ES2	2	p53 activity restoration	chemotherapy, ageing, lung fibrosis	[27–29]
HSP90 inhibitors	alvespimycin, ganetespib	2	AKT downregulation	ageing	[30]
BET degraders and inhibitors	ARV-825, JQ1	3	autophagy (ferroptophagy) activation,ferroptosis, NHEJ inhibition	obesity, chemotherapy	[31,32]
cardioglycosides	ouabain, digoxin, strophanthidin, bufalin	3	autophagy inhibition, modulation of transmembrane potential and intracellular pH	chemotherapy, lung fibrosis, pre-neoplastic lesions, ageing	[33–35]
fibrates	fenofibrate	3	autophagy activation	—	[36]
autophagy blockers	chloroquine, bafilomycine A1	3	autophagy inhibition	chemotherapy	[14,34]
glutaminolysis inhibitors	BPTES	3	gutaminolysis inhibition, acidosis	ageing	[37]
piperlongumine	—	3	OXR1 inhibition, ROS production	—	[38,39]

Cellular senescence is largely regarded as a cell fate in response to stress, characterized primarily by a highly stable proliferative arrest associated with the increased expression of some cyclin-dependent kinase inhibitors (e.g. p16, p15 and p21), and often accompanied by a complex secretome termed the senescence-associated secretory phenotype (SASP). Other non-obligatory, though often encountered senescence hallmarks, include senescence-associated β-galactosidase activity (SA-βGal) resulting from increased lysosomal content [40], resistance to apoptosis, persistent DNA damage foci, modifications of chromatin and decreases in lamin-B [41]. The only universal feature shared by all types of senescent cells is stable withdrawal from the cell cycle, though this is not exclusive to senescence as terminal differentiation and T cell replicative exhaustion also involve a durable proliferative arrest. Indeed, it remains unclear whether or not some terminally differentiated cells may become senescent. In this review, we propose a comprehensive conceptualization of senolysis in three possible routes, with an emphasis on the selectivity of senolytic drugs for different types of senescent cells, thus showcasing the diversity of senescent phenotypes.

Senolytics can be classified in at least three categories, as they target senescent cells at one of three levels. Class I senolytics target directly BCL-2 family proteins, which in senescence are rearranged in a network distinct from that of non-senescent cells, resulting in dependency on anti-apoptotic BCL-2 family members for survival; class II senolytics target upstream pathways that provide senescent cells with resistance to cell death, such as the USP7/MDM2/p53 axis, or AKT pro-survival signalling; and class III senolytics further disturb homeostatic processes that are already dysregulated in senescent cells, such as proteostasis maintenance or redox homeostasis.

## 2. Class I senolytics: directly targeting apoptosis gatekeepers

Commitment to apoptosis is directly controlled by the stoichiometry of BCL-2 family proteins, or BH3 proteins, that differentially interact with each other owing to their BCL-2 homology domains BH1–4 [42,43]. Three distinct classes of BCL-2 family proteins can be distinguished, classified according to the BH domains they contain. Pro-apoptotic BH1–3 effectors BAX and BAK oligomerize to drive mitochondrial outer membrane permeabilization (MOMP), which is the irreversible tipping point leading to cell death orchestration. In unstressed conditions, MOMP is prevented by the sequestration of BH1–3 factors by anti-apoptotic BH1–4 guardians such as BCL-2, BCL-xL or MCL-1. Finally, upstream pro-death or pro-survival signals translate into the modulation of the activity of BH3-only apoptotic inducers, such as NOXA, PUMA or BIM. BH3-only proteins can either act as sensitizers that sequester pro-survival BH1–4 factors, or as activators by directly interacting with BAX/BAK and catalytically favouring their oligomerization [44,45]. Due to genetic variability in their BH3 sequences, individual members of each class have their own profile of interactions with other BCL-2 family proteins in terms of affinity and selectivity, forming a complex but organized and finely tuned BH3 network [46].

**Figure 1.**
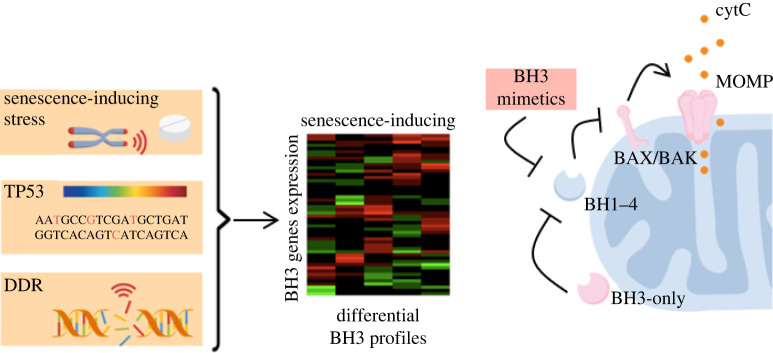
Class I senolytics target apoptosis-primed BH3 networks of senescent cells. In response to stress, the cell fate decision to overgo senescence rather than apoptosis is accompanied by the reorganization of BH3 networks and the apoptotic priming of senescent cells. BH3 profiles evolve dynamically during senescence onset and are dictated by the nature of the senescence-inducing stressor and its magnitude, expression levels and mutational status of TP53, as well as the establishment or lack thereof of a DNA damage response. The reorganization of BH3 networks in senescent cells renders them sensitive to the action of BH3 mimetics that bind anti-apoptotic BH1–4 proteins to increase the apparent stoichiometry of pro-apoptotic BH3-only proteins and promote BAX/BAK oligomerization and MOMP.

BCL-2 family proteins and notably BH1–4 anti-apoptotic guardians are essential in orienting cell fate towards senescence by suppressing apoptosis in response to stress. Commitment of triple-negative breast cancer cells to senescence rather than apoptosis in response to treatment with BET inhibitors was found to be largely determined by the upregulation of BCL-xL [47]. In different contexts of p53 activation, BCL-2 and MCL-1 were found to promote growth arrest over cell death [48,49]. MCL-1 upregulation also promoted survival and senescence in IMR90 fibroblasts following aberrant mitosis caused by RASval12 expression [50]. However, cell death avoidance in the favour of senescence comes at the cost of the apoptotic priming of senescent cells, through the formation of stable complexes between BH1–4 proteins and pro-apoptotic BH3-only factors. The so-called one-two punch approach consists in suppressing tumours by first inducing senescence in cancer cells by the means of radiotherapy or chemotherapy and then eliminating now-senescent cancer cells with senolytic compounds. The reorganization of the BH3 network in senescent cells sensitizes them to so-called BH3 mimetics [51–53] (figure 1). BH3 mimetics are small synthetic compounds that mimic the BH3 domain of pro-apoptotic BH3-only inducers. They bind to and inhibit anti-apoptotic BH1–4 guardians and prevent them from interacting with other BCL-2 family proteins, thus increasing the apparent stoichiometry of BH3-only proteins and shifting the equilibrium towards BAX/BAK oligomerization and apoptosis. The senolytic potential of BH3 mimetics was predicted from transcriptomic analyses revealing an increased expression of BH1–4 anti-apoptotic factors in senescence [51,52]. Navitoclax is by far the most studied BH3 mimetic senolytic. It inhibits BCL-2, BCL-xL and BCL-w. It is considered to be a broad-spectrum senolytic, as it is efficient against a large panel of senescent cells [54]. Inhibition of BCL-xL, but not BCL-2, was required for navitoclax-mediated senolysis of breast and lung cancer cells induced in senescence by genotoxic agents etoposide and doxorubicin [55], and irradiation-induced senescent meningioma cells [56]. Interestingly, prostate cancer cells were killed by navitoclax or BCL-xL-specific inhibitors if induced in senescence by irradiation or genotoxic agents, but not if the proliferative arrest was triggered by antiandrogen enzalutamide, which does not damage DNA [57]; the proliferative arrest induced by enzalutamide was however reversible upon withdrawal of the drug, calling into question the senescent state of these cells. Other reports of navitoclax-resistant cancer cells induced in senescence by non-genotoxic chemotherapeutic agents such as alisertib or palbociclib [33] suggest that the DNA damage response may be important in reorganizing BCL-2 family factors into a navitoclax-sensitive BH3 network in senescent cancer cells. It is known that the DNA damage response differentially regulates the expression of BCL-2 family members [58]. TP53 mutational status may also affect the sensitivity of DNA damage-induced senescent cancer cells to BH3 mimetics, as the regulation of the expression of some BCL-2 family genes was found to be p53-dependent in response to genotoxicity [59,60]. Navitoclax resistance in wild-type TP53 therapy-induced senescent breast cancer cells expressing low levels of NOXA was overcome by dual treatment with a specific MCL-1 inhibitor [17]. Interestingly in this study, sensitivity to BCL-xL or BCL-xL/MCL-1 inhibition depended primarily on the cell line and was largely conserved for various senescence-inducing insults. This is consistent with recent work demonstrating that in senescent cancer cells, gene expression dynamics, SASP composition and sensitivity to BH3 mimetics correlated more with the cell type than with the nature of the senescence-inducing stressor [61].

There are conflicting results on the ability of navitoclax to target etoposide-induced senescent primary lung fibroblasts. IMR90 cells exposed for 48 h to 20 µM etoposide to induce senescence were killed by 2.5 µM navitoclax when added for 48 h after a 2-day etoposide withdrawal [36], whereas 10 µM navitoclax did not affect cell viability if added for 48 h after a longer 6-day etoposide withdrawal [1]. This may be explained by a dynamic evolution of BCL-2 protein levels during senescence onset, from a navitoclax-sensitive to insensitive BH3 network. Furthermore, if IMR90 cells were induced in senescence with a higher concentration of 50 µM etoposide for 48 h followed by a 5-day withdrawal, a 72 h exposure to as little as 1 µM navitoclax was senolytic [33]. This in turn suggests that beyond the cell type and the nature of the senescence-inducing insult, the magnitude of the stress may also influence the resulting evolution of BH3 profiles that underlies sensitivity to BH3 mimetics, possibly through the DNA damage response activation level. Similarly, while early senescent glioblastoma cells were killed by selective BCL-2 inhibition—although no proliferating control cells were included in the assay [62], late senescent glioblastoma cells were insensitive to BCL-2 inhibition and depended solely on BCL-xL for their survival [63]. In IMR90 cells, when compared to levels in proliferation, BCL-2, BCL-xL and BCL-w proteins all displayed a marked increase in etoposide and replicative senescence, whereas this increase was more important for BCL-2 than for BCL-xL and BCL-w in RASval12 senescence [51]. This may explain the efficiency of the specific BCL-2 inhibitor venetoclax in RASval12-induced senescent IMR90 only, while it appeared necessary to inhibit all BCL-2, BCL-xL and BCL-w with navitoclax or the related compound ABT-737 to kill etoposide- and replicative-senescent IMR90.

Neither navitoclax nor BCL-xL-specific inhibitors A1331852 and A1155463 are senolytic towards irradiation-senescent preadipocytes [52,64]. Upon senescence induction, whereas navitoclax- and A1331852/A1155463-sensitive human umbilical vein endothelial cells (HUVECs) and IMR90 fibroblasts showed a marked increase in BCL-xL and BCL-2 protein levels, preadipocytes exhibited steadier BCL-xL and even decreased BCL-2 protein levels, in contrast with significantly increased BCL-w protein levels [52]. Senescent preadipocytes may then rely preferentially on BCL-w for their survival. Although none is available at the moment [65], selective BCL-w inhibitors should be developed since they are expected to be toxic for senescent preadipocytes. Preadipocytes may be among the most abundant senescent cell types in old age and mediate age-related metabolic disorders, making them targets of interest [66].

The sensitivity of senescent cells to different BH3 mimetics correlated well with the expression or protein levels of the various anti-apoptotic BCL-2 family factors; however, the levels of pro-apoptotic effectors and initiators were rarely assessed. This is unfortunate, because the potency of a given BH3 mimetic as a senolytic does not depend solely on the protein levels of its BH1–4 targets, but rather on the balance between these and their pro-apoptotic partners [67]. In a panel of soft-tissue sarcoma cell lines induced into senescence by irradiation, BCL-2 or BCL-xL were differentially increased depending on the cell line, but the cells were all comparably sensitive to senolysis by venetoclax or navitoclax, irrespectively of BCL-2 and BCL-xL expression levels [68]. Similarly, while irradiation-induced senescent WI-38 fibroblasts upregulated BCL-xL and BAK but not BCL-2, they were only sensitive to dual BCL-xL/BCL-2 inhibition, but not to either factor alone [54]. Therefore, a more exhaustive characterization of BH3 networks in senescence models could guide the choice for better class I senolytics, for example through the BH3 profiling method [69–72], which could significantly improve selectivity prediction of BH3 mimetics as senolytics.

Both navitoclax and ABT-737 were efficient senolytics in multiple preclinical models. Nevertheless, their translation into clinic as senolytics is impaired by their reported toxicity towards platelets and neutrophils due to the targeting of BCL-xL and BCL-2, respectively, leading to thrombocytopenia and neutropenia [73,74]. Given the high potential of BH3 mimetics as senolytics, various strategies are being designed to overcome this issue and improve their *in vivo* tolerability and therapeutic window, including conjugation of navitoclax to galactose for its specific release in senescent cells due to senescence-associated β-galactosidase activity [75], synthesis of BCL-xL proteolysis-targeting chimera (PROTACs) taking advantage of low E3 ligase expression in platelets [76] and galactose-functionalized nanoparticle encapsulation [77]. In the future, inhibitor of apoptosis (IAP)-based PROTACs could also hold promise as senolytics [78], especially in the light of recent work reporting on the overexpression of some IAP members in senescence [62].

## 3. Class II senolytics: modulating upstream pro-survival pathways

**Figure 2.**
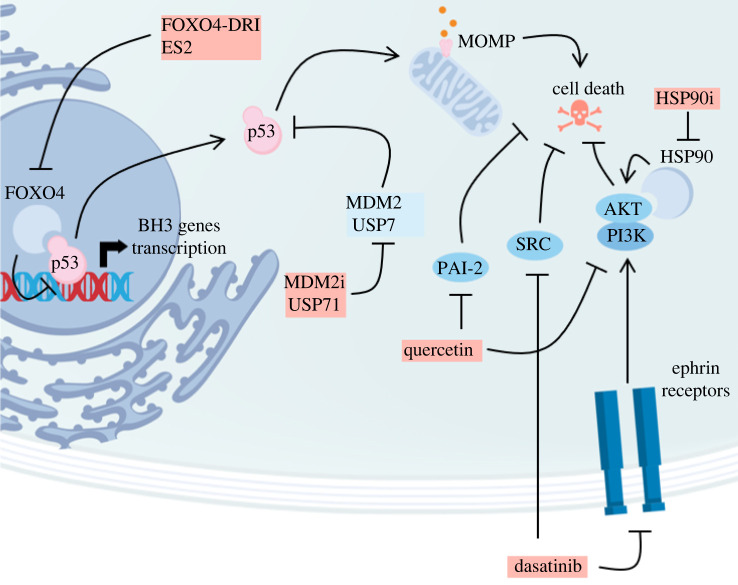
Class II senolytics inhibit survival pathways elicited by senescent cells. Senescent cells promote survival through the downregulation of p53 activity via its nuclear segregation by FOXO4. Senolytic peptides FOXO4-DRI and ES2 suppress the FOXO4-p53 interaction and promote p53 nuclear exclusion and the subsequent triggering of apoptosis. Besides p53 levels are actively kept low in senescent cells through MDM2 and USP7, the inhibition of which also results in apoptosis. The dasatinib and quercetin senolytic cocktail targets multiple survival-associated pathways including ephrins, PAI-2, SRC and AKT signalling. In senescent cells, AKT signalling is sustained through the stabilization of phosphorylated AKT by HSP90 chaperones. HSP90 inhibitors downregulate AKT and induce senolysis.

The SASP secreted by some senescent cells comprises apoptosis-promoting factors as well as pro-inflammatory factors and proteases, together resulting in a harsh microenvironment [15,79,80]. Independently of the reorganized BH3 networks, senescent cells also resist cell death through the upregulation of upstream pro-survival signalling pathways that can be targeted for senolysis (figure 2).

Classically, p53 is transiently upregulated in response to stress to trigger senescence-associated cell cycle exit through the transcription of p21 and then returns to lower cellular levels to maintain the proliferative arrest [81] and to participate in the regulation of other aspects of senescence such as SASP expression [82–84]. In addition to its functions as a cell–cycle inhibitor, p21 also has anti-apoptotic activity in senescent cells [85]. However, when expressed at high levels, p53 can also promote apoptosis by both upregulating the transcription of BH3-only genes and interacting with BCL-2 family proteins in the cytosol and at the mitochondrial outer membrane [44]. In cells with established senescence, p53 levels are maintained low through ubiquitination and its pro-apoptotic activity is repressed through nuclear segregation [21,27]. Thus, the restoration of p53 activity by either nuclear exclusion or suppression of its ubiquitination can lead senescent cells to apoptosis.

Transcription factor FOXO4 is upregulated in senescence to participate in proliferative arrest maintenance, and it physically interacts with p53 in.the nucleus [86–88]. This interaction both prevents p53 from inducing the transcription of pro-apoptotic target genes and restricts p53 localization to the nucleus so that it cannot interact with BCL-2 family proteins in the cytosol and at the mitochondrial outer membrane [27,28]. The senolytic peptide FOXO4-DRI, by binding p53 and relieving the p53-FOXO4 interaction, abrogated the nuclear sequestration of p53 that consequently migrated towards the cytosol and the mitochondria, where it triggered apoptosis [27]. A computationally designed peptide, ES2, binds FOXO4 rather than p53, and like FOXO4-DRI, induced the nuclear exclusion of p53 and selectively elicited the death of senescent cells *in vitro* and *in vivo* [28]. Remarkably, the occurrence of the p53-FOXO4 nuclear interaction and the senolytic potential of its disruption was recapitulated in many different senescent models [27,28,89,90]. The development of small synthetic compounds disrupting the p53-FOXO4 interaction is thus of interest to exploit this senolysis mechanism in a therapeutic context, as small molecule compounds bear more favourable pharmacokinetics properties than peptides [91].

p53 protein levels are largely controlled and kept low by proteasomal degradation due to ubiquitination by the MDM2 E3 ligase. The stability of MDM2 is itself enhanced through its deubiquitination by USP7. Therefore, both USP7 and MDM2 negatively regulate the stability of p53 [92]. As predicted, USP7 inhibitors restored p53 activity in senescent cells and selectively triggered senescent cell death [21]. Surprisingly, USP7 inhibitors increased p53 levels in senescent cells, but not in proliferative cells, which suggests a higher dependency of senescent cells on the USP7/MDM2 axis for the regulation of p53 activity compared to their proliferative counterparts. Downstream of USP7, directly inhibiting MDM2 was also senolytic, but USP7 inhibitors reportedly exhibit fewer side-effects than MDM2 inhibitors in mice [21,22]. However, the proprietary MDM2 inhibitor UBX0101 failed a phase II clinical trial as a senolytic in patients with osteoarthritis [93]. Interestingly, the perturbation of the BH3 network and restoration of p53 activity appear to synergize to trigger senolysis, as a combination of navitoclax with USP7 or MDM2 inhibitors was more potent than either treatment alone [21,23]. Recent work in cancer cells showed that apoptotic priming of BH3 networks favoured cell death in response to restoration of p53 activity [94].

Moving away from p53, AKT signalling participates in the survival of many senescent cells. AKT is a pleiotropic serine/threonine protein kinase that reportedly regulates over 100 downstream substrates, playing a central role in a complex network of signalling pathways comprising multiple positive and negative feedback loops. AKT promotes survival notably through the inhibition of BH3-only protein BAD, the downregulation of p53 via MDM2 and the upregulation of anti-apoptotic BH1–4 protein MCL-1 [95,96]. Transcriptome analysis of irradiation-induced senescent preadipocytes highlighted the upregulation of various survival-associated pathways including ephrin-B-dependent suppression of apoptosis and the PI3K/AKT pathway that can be targeted by dasatinib and quercetin, respectively [15]. Dasatinib is a broad-spectrum tyrosine kinase inhibitor that notably targets, besides SRC, various ephrin receptors that promote survival via stimulation of AKT signalling [97,98]. Dasatinib as a senolytic is widely used in combination with quercetin, which targets notably PI3K and PAI-2. PI3K is directly activated by ephrin receptors, so quercetin can further participate in the dasatinib-induced downregulation of AKT signalling. On the other hand, pro-survival effects of PAI-2 are known but poorly understood. PAI-2 inhibition can result in the destabilization of p21, leading to apoptosis [99]. Furthermore, PAI-2 inhibition results in the transcriptional activation of E2F-regulated pro-apoptotic genes due to Rb destabilization [100]. While the senolytic activity of dasatinib was assigned to ephrin receptor inhibition, recent work demonstrated that SRC was an essential factor favouring survival and senescence over apoptosis in response to genotoxicity, through the downregulation of p53 [101]. It is therefore plausible that dasatinib triggers senolysis in part through inhibition of survival-associated SRC signalling, even though, to date, no senolysis by direct SRC inhibition has been demonstrated.

Senolysis by HSP90 inhibitors is thought to be mediated in part by the downregulation of AKT signalling. HSP90 chaperone proteins increased the stability of active, phosphorylated AKT, reinforcing pro-survival signalling in senescent cells. Inhibiting HSP90 chaperones led to the destabilization and degradation of active AKT and other client proteins. In oxidative stress-induced senescent MEFs, HSP90 inhibitors were senolytic, whereas specific AKT inhibitors were not. However, quercetin which targets other pathways in addition to PI3K/AKT was senolytic in this model [30], suggesting that the essentiality of HSP90 proteins in senescence is imputable to the stabilization of not only AKT but other pro-survival factors. Directly targeting AKT with inhibitor MK2206 was senolytic in enzalatumide-induced senescent prostate cancer cells, but it induced senescence in their non-senescent counterparts [102].

Overall, many types of senescent cells rely at least partially on upregulated AKT signalling for apoptosis suppression, and upstream targeting of this pathway at the levels of ephrin receptors, PI3K or HSP90 chaperones, often with the concomitant inhibition of parallel survival axes, proved efficient approaches for the selective clearance of senescent cells. Nevertheless, downregulating AKT is not always sufficient to induce senolysis. Dasatinib was senolytic in preadipocytes but not in HUVECs, which was consistent with the fact that the former but not the latter was sensitive to siRNA-mediated knockdown of ephrin genes. Conversely, quercetin which targets PI3K and PAI-2 was senolytic in HUVECs but not in preadipocytes [15]. The related flavonoid fisetin exhibited the same senolytic selectivity [64]. This suggests that while senescent preadipocytes rely primarily on ephrin signalling for survival, the inhibition of PAI-2 anti-apoptotic mechanisms is required to induce apoptosis of senescent HUVECs, in which targeting the PI3K/AKT axis is not sufficient to trigger senolysis. HSP90 inhibitors that downregulate AKT were not senolytic either in irradiation-induced senescent preadipocytes [30]. This could indicate that the senolytic effect of dasatinib in these cells was mediated by SRC inhibition rather than the suppression of PI3K/AKT signalling, on which senescent preadipocytes do not seem to rely for survival. Whereas AKT activity is upregulated in many senescence models, it was shown to be reduced to levels even below those of proliferative cells in some forms of oncogene-induced senescence (OIS) [103]. Accordingly, HSP90 inhibitors and dasatinib were not senolytic in BRAF-V600E-induced senescent fibroblasts [34]. Downstream of AKT, inhibiting mTOR was senolytic in liver cancer cells induced in senescence by CDC7 inhibition, but not in OIS [104]. Although the lack of PI3K/AKT hyperactivity could explain the resistance of certain senescent cells to senolytics directly or indirectly targeting this pathway, more complex regulation and crosstalk are certainly at play.

## 4. Class III senolytics: further disturbing cellular homeostatic processes

**Figure 3.**
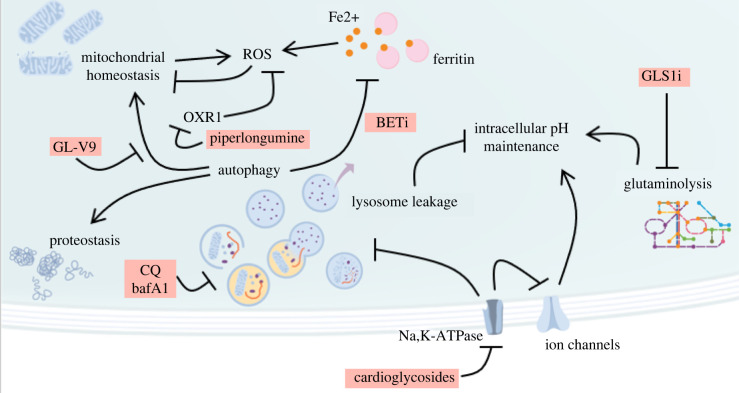
Class III senolytics disrupt homeostatic processes already challenged in senescent cells. Multiple facets of homeostasis are interconnected in senescence and regulated differently than in proliferative cells. Autophagy is essential for proteostasis maintenance as well as mitochondrial homeostasis through mitophagy, which is inhibited by GL-V9. Mitochondrial dysfunction in senescent cells leads to the formation of ROS. Redox stress responses may be orchestrated by OXR1, which is inhibited by piperlongumine. The inhibition of ferritinophagy by BET inhibitors leads to ferroptosis and ROS formation. Intracellular pH of senescent cells is reportedly acidified by lysosome leakage, which is compensated for by ammonia production through glutaminolysis. Inhibitors of glutaminase 1 induce acidosis and are thus senolytic. Cardioglycosides, by binding to the Na,K-ATPase, both alter membrane potential and intracellular pH regulation of senescent cells, as well as inhibit autophagy through signal transduction.

The last level on which senescent cells can be targeted for elimination is through further disturbance of cellular homeostatic processes that are already dysregulated in senescence, often in a systemic manner, such as proteostasis, or mitochondrial and redox homeostasis (figure 3).

Proteostasis results from a tightly regulated balance between protein synthesis, folding and degradation that are coordinated to ensure a functional proteome and proper cell function. Mitochondrial homeostasis, redox homeostasis and global proteostasis all influence each other. Proteostasis decline and mitochondrial dysfunction are hallmarks of ageing and cellular senescence. Failure to restore proteostasis in the face of proteotoxic insults triggers senescence, as shown in keratinocytes in response to advanced glycation end products formation [105], or in post-mitotic neurons facing increasing proteotoxicity during long-term culture [106]. Replicative-senescent fibroblasts experience a global decline in proteostasis, with dysregulated alternative splicing, and altered responses to proteotoxic stress including disruptions in the heat shock response and the unfolded protein response (UPR) [107]. Aged mice exhibit mild chronic endoplasmic reticulum (ER) stress and UPR activation, and trigger an exaggerated sustained response lacking fine tuning when subjected to further ER stress [108]. Senescent cells with ample SASP synthesis may mitigate proteotoxicity and the burden on their secretory systems by chronically upregulating the ubiquitin/proteasomal axis and the autophagy/lysosomal axis, and differentially activating UPR branches [14,109]. Meanwhile, improved protein translation fidelity results in extended lifespan in several model organisms [110]. Different facets of cellular homeostasis share common regulators in ageing. Transcription factor MondoA was found to delay replicative and DNA damage-induced senescence through the downregulation of autophagy suppressor Rubicon as well as maintenance of mitochondrial redox homeostasis through Prdx3 expression [111].

The facet of proteostasis that is the most targeted so far by known senolytics is autophagy. Autophagy is an adaptive cellular process in response to stress or energy deprivation, through which organelles and proteins are degraded (specifically as in selective autophagy, or non-specifically as in bulk autophagy) and broken down to small metabolic substrates that are recycled to maintain essential biosynthetic activities. Autophagy is first and foremost a survival process, but unimpeded autophagy can lead to apoptosis, necrosis, or even autophagic cell death in which the cell, overwhelmed by the presence of autolysosomes, is ‘eating itself to death’ [112]. Relationships between autophagy and senescence are seemingly paradoxical and yet to be fully understood. Basal autophagy in proliferative cells is considered anti-senescent because it decreases the cellular burden of potential senescence-inducing stressors, thereby acting as a preferred primary stress response. In this regard, inhibiting autophagy induces senescence in glioblastoma and bronchial epithelial cells [113,114]. Downregulating autophagy master regulator ULK1 induces senescence in a wide range of cancer cells and sensitizes them to navitoclax-induced apoptosis [115]. However, in some contexts of high-intensity cellular stress, autophagy rather suppresses apoptosis and promotes senescence induction [116]. Autophagy is especially important during OIS onset: inhibiting autophagy delays entry into senescence and even allows the full bypass of BRAF senescence in melanocytes [117]. Since senescent cells maintain high levels of protein, autophagy presumably contributes to proteostasis by preventing unlimited cell growth in the absence of cell proliferation. Autophagy also clears macromolecules damaged from increased metabolic fluxes and ROS production [118]. Therefore, in low to mild stress conditions, autophagy suppresses senescence by mediating the return to homeostasis and proliferation, whereas in the face of higher intensity stress, autophagy favours senescence by suppressing apoptosis. Beyond bulk autophagy, the degradation of specific factors through selective autophagy via various ATG8 family receptors was shown to be essential in the homeostatic maintenance of both replicative and DNA damage-induced senescence [119]. Selective autophagy of KEAP1, TNIP1 and NDP52 regulated redox homeostasis, SASP expression and proteostasis, respectively, in senescent cells. Further exploration of selective autophagy networks in various types of senescent cells may foster the development of new-generation precision senolytics targeting autophagy, with improved proliferative versus senescent and inter-senescent selectivity.

Most senolytics that affect autophagy are inducers of the process. HSP90 inhibitors were identified in the rational screening of a small chemical library of compounds targeting autophagy, of which all senolytic hits were autophagy inducers [30]. BET inhibitors are another group of senolytic drugs that exert their action through pleiotropic effects including the upregulation of autophagy and the attenuation of non-homologous end-joining repair [31]. They proved efficient in mice models of obesity and chemotherapy-induced senescence. Their senolytic action was later assigned to the selective autophagy of ferritin (ferritinophagy) and the subsequent triggering of non-apoptotic, iron-dependent cell death ferroptosis, at least in therapy-induced senescent fibroblasts [32]. Indeed, replicative and irradiation-induced senescent fibroblasts and epithelial cells accumulate important amounts of intracellular iron, through the combined upregulation of iron-storage protein ferritin expression and inhibition of ferritinophagy [120]. PPARα agonist fenofibrate was senolytic through autophagy upregulation in IL-6-induced senescent chondrocytes [36], but in TNFα-induced senescent synovial fibroblasts, it attenuated the senescent phenotype and inhibited SASP expression without exhibiting a differential toxicity towards senescent cells, a behaviour corresponding to so-called senomorphic compounds [121].

Another senolytic strategy consists in depriving senescent cells from cytoprotective autophagy. In the first demonstration of selective elimination of senescent cells through pharmacological means, therapy-induced senescent lymphoma cells were shown to upregulate autophagy to cope with ER stress resulting from SASP production. The blockade of autophagy flux with bafilomycin A1 led to senolysis [14]. Autophagy flux also increased in BRAF-V600E OIS fibroblasts, and inhibiting autophagy with chloroquine, bafilomycin A1, or low concentrations of cardioglycosides inducing Na,K-ATPase signal transduction and notably AKT, resulted in the specific senolysis of BRAF-senescent cells [34]. The essentiality of autophagy in some OIS models may correlate with the downregulation of basal AKT signalling in these cells. Whereas many senescent cells have upregulated AKT signalling and are sensitive to autophagy-inducing senolytics, in some instances OIS cells rather downregulate AKT signalling and increase basal autophagy, as concomitant oncogene and AKT activation favours senescence bypass and transformation [103,122,123].

Besides proteostasis and autophagy, other cellular homeostatic processes differentially regulated in senescence can be targeted for senolysis. Aforementioned cardioglycosides are broad-spectrum senolytics that target a myriad of senescent cell types. They proved efficient in mouse models of OIS, chemotherapy-induced senescence, natural ageing and irradiated mice [33]. They bind the transmembrane Na,K-ATPase pump, which is involved in both membrane potential maintenance and signal transduction. While low doses of cardioglycosides induced the selective senolysis of BRAF-V600E-induced senescent fibroblasts through signal transduction and autophagy inhibition [34], their action in other senescence models was rather attributed to disturbance of membrane potential [33,35]. Interestingly, they proved inefficient in senescent human mesenchymal stem cells that have upregulated processes for potassium import but could nevertheless be primed for senolysis through the inhibition of anti-apoptotic MCL-1 [124]. On the other hand, the authors noted that etoposide-induced senescent A549 cells exhibited a decreased capacity to restore intracellular potassium levels, the drop of which is an early event in apoptosis, and as such made them less resilient in the face of stress, contradicting the notion of senescence-associated apoptosis resistance. Differential sensitivity between senescent and normal cells upon Na,K-ATPase pump inhibition was also attributed to the modification of intracellular pH [35]. Glutaminolysis inhibition was another senolytic strategy relying on differences in intracellular pH regulation between senescent and normal cells. Senescent cells were found to undergo intracellular acidosis because of lysosomal leakage and to rely on increased glutaminolysis-produced ammonia to neutralize their intracellular pH. Inhibiting glutaminase 1 consequently led to the selective clearance of senescent cells [37].

Redox and mitochondrial homeostasis are intimately linked in senescence. Mitochondrial mass increases in senescence, but this is accompanied by mitochondrial dysfunction and the production of ROS, generating chronic oxidative stress [125,126]. ER-mitochondria contact sites appear critical in senescence regulation [127], and mitochondrial dysfunction and ROS production were shown to be mediated in many senescence models by increased IP3R-mediated mitochondrial calcium uptake from the ER following ITPR downregulation [128]. Oxidative stress induces senescence in many settings [129,130], but the upregulation of ROS detoxifying systems is also critical for the decision to undergo senescence over apoptosis [126]. Consequently, targeting these detoxifying systems in established senescent cells can yield senolysis. The alkalization of lysosomes by GL-V9 was shown to impair mitophagy and further increase dysfunctional mitochondrial mass in senescent cells, leading to ROS overproduction and apoptosis [131]. Piperlongumine is another senolytic causing redox imbalance in senescent cells through the targeting of oxidative stress sensor OXR1 [38,39]. Procyanidin C1 was senomorphic at lower concentrations and senolytic at higher concentrations, and acted by further promoting ROS formation in senescent cells, leading to mitochondrial dysfunction [132].

Still how these various homeostatic processes are affected depending on senescent cell type largely remains to be explored. Mapping their interconnections, for example, through the identification of common regulators like MondoA affecting both autophagy/proteostasis and mitochondrial function/redox homeostasis [111], shall yield novel avenues for selective senolysis.

## 5. Conclusion and perspectives

Throughout this review, we proposed a conceptualization of senolysis based on mechanisms of action. This approach highlights the compelling diversity of senescent phenotypes in terms of apoptotic priming, cell death-suppressing strategies and homeostatic regulation. The only universal, though not exclusive, feature of senescent cells is an irreversible or highly stable proliferative arrest, but even this is being challenged, as various stressors can induce so-called senescent-like states of reversible proliferative arrest that exhibit classical senescence hallmarks such as senescence-associated β-galactosidase activity, SASP expression and even sensitivity to senolytics [57,133]. We argue that taking into account the heterogeneity of senescent phenotypes would stimulate context-based senolytic drug development, yielding highly potent candidates more selective towards a subset of senescent cells. This is especially relevant considering the increasing awareness of the fact that indiscriminate, systemic removal of senescent cells may be harmful, as some senescent cell subpopulations appear to be beneficial [134–136]. Inter-senescent cell selectivity of senolytic compounds, rather than being a limitation of senolytic therapy, thus actually constitutes an opportunity for precision clearance of specific subsets of senescent cells in defined pathological contexts.

Innovative strategies like galacto-conjugation [137] or encapsulation in galactose-functionalized silica beads [138] can be used to bypass limitations caused by adverse side-effects of some drugs, such as BH3 mimetics. These strategies can also turn toxic molecules that do not discriminate between senescent and non-senescent cells into bona fide senolytics, through their release in senescent cells only. Nevertheless, non-senescent cells expressing high levels of β-galactosidase would also in principle be sensitive to their effects.

Mechanistic insights are lacking for some promising senolytic candidates that could lead to the identification of senolytic targets and the development of even more potent compounds. Curcumin analogue EF24 is a senolytic in several models and induces the proteasomal degradation of BH1–4 anti-apoptotic factors via an unknown route [139]. It is however unclear whether curcumin, which itself is a controversial lead [140,141], and the related compound o-vanillin, are bona fide senolytics or rather function as senomorphics [142,143]. Similar uncertainties exist about fisetin [64,144]. This highlights the need, especially *in vivo*, for unequivocally characterizing senolysis over senomorphism, which can be undesirable if it allows the reentrance of pre-neoplastic cells into the cell cycle. In vivo characterization of senolysis is now facilitated by the detection of a senescence-specific oxylipin released upon lysis of senescent cells [145]. It also remains a prime consideration to investigate and understand in details the molecular mechanisms underlying senolysis by novel compounds, notably by identifying with certainty the cellular target through which the compound exerts its activity and the signalling pathways leading to cell death, which has sometimes been neglected. Systematically characterizing the mechanisms of action of senolytics would yield opportunities to either develop optimized compounds for the identified target, or to discover even more interesting targets for senolysis within the same pathway.

## Data Availability

This article does not include any original or unpublished research or data.

## References

[1] Schafer MJ et al. 2017 Cellular senescence mediates fibrotic pulmonary disease. Nat. Commun. **8**, 14532. (10.1038/ncomms14532) 28230051 PMC5331226

[2] Pan J et al. 2017 Inhibition of Bcl-2/xl with ABT-263 selectively kills senescent type II pneumocytes and reverses persistent pulmonary fibrosis induced by ionizing radiation in mice. Int. J. Radiat. Oncol. Biol. Phys. **99**, 353-361. (10.1016/j.ijrobp.2017.02.216) 28479002 PMC6853175

[3] Wiley CD et al. 2019 Secretion of leukotrienes by senescent lung fibroblasts promotes pulmonary fibrosis. JCI Insight **4**, e130056. (10.1172/jci.insight.130056) 31687975 PMC6975274

[4] Thompson PJ, Shah A, Ntranos V, Van Gool F, Atkinson M, Bhushan A. 2019 Targeted elimination of senescent beta cells prevents type 1 diabetes. Cell Metab. **29**, 1045-1060.e10. (10.1016/j.cmet.2019.01.021) 30799288

[5] Palmer AK et al. 2019 Targeting senescent cells alleviates obesity-induced metabolic dysfunction. Aging Cell **18**, 1-15. (10.1111/acel.12950) PMC6516193 30907060

[6] Sierra-Ramirez A, López-Aceituno JL, Costa-Machado LF, Plaza A, Barradas M, Fernandez-Marcos PJ. 2020 Transient metabolic improvement in obese mice treated with navitoclax or dasatinib/quercetin. Aging (Albany NY) **12**, 11 337-11 348. (10.18632/aging.103607) PMC7343475 32584785

[7] Bussian TJ, Aziz A, Meyer CF, Swenson BL, van Deursen JM, Baker DJ. 2018 Clearance of senescent glial cells prevents tau-dependent pathology and cognitive decline. Nature **562**, 578-582. (10.1038/s41586-018-0543-y) 30232451 PMC6206507

[8] Musi N, Valentine JM, Sickora KR, Baeuerle E, Thompson CS, Shen Q, Orr ME. 2018 Tau protein aggregation is associated with cellular senescence in the brain. Aging Cell **17**, e12840. (10.1111/acel.12840) 30126037 PMC6260915

[9] Zhang P et al. 2019 Senolytic therapy alleviates Aβ-associated oligodendrocyte progenitor cell senescence and cognitive deficits in an Alzheimer's disease model. Nat. Neurosci. **22**, 719-728. (10.1038/s41593-019-0372-9) 30936558 PMC6605052

[10] Ogrodnik M et al. 2021 Whole-body senescent cell clearance alleviates age-related brain inflammation and cognitive impairment in mice. Aging Cell **20**, 1-16. (10.1111/acel.13296) PMC7884042 33470505

[11] Childs BG, Baker DJ, Wijshake T, Conover CA, Campisi J, Deursen JV. 2016 Senescent intimal foam cells are deleterious at all stages of atherosclerosis. Science **354**, 472-477. (10.1126/science.aaf6659) 27789842 PMC5112585

[12] Childs BG, Zhang C, Shuja F, Sturmlechner I, Trewartha S, Velasco RF, Baker DJ, Li H, van Deursen JM. 2022 Senescent cells suppress innate smooth muscle cell repair functions in atherosclerosis. Nat. aging **1**, 698-714. (10.1038/s43587-021-00089-5) PMC8570576 34746803

[13] Roos CM et al. 2016 Chronic senolytic treatment alleviates established vasomotor dysfunction in aged or atherosclerotic mice. Aging Cell **15**, 973-977. (10.1111/acel.12458) 26864908 PMC5013022

[14] Dörr JR et al. 2013 Synthetic lethal metabolic targeting of cellular senescence in cancer therapy. Nature **501**, 421-425. (10.1038/nature12437) 23945590

[15] Zhu Y et al. 2015 The Achilles' heel of senescent cells: from transcriptome to senolytic drugs. Aging Cell **14**, 644-658. (10.1111/acel.12344) 25754370 PMC4531078

[16] Kirkland JL, Tchkonia T. 2015 Clinical strategies and animal models for developing senolytic agents. Exp. Gerontol. **68**, 19-25. (10.1016/j.exger.2014.10.012) 25446976 PMC4412760

[17] Shahbandi A, Rao SG, Anderson AY, Frey WD, Olayiwola JO, Ungerleider NA, Jackson JG. 2020 BH3 mimetics selectively eliminate chemotherapy-induced senescent cells and improve response in TP53 wild-type breast cancer. Cell Death Differ. **27**, 3097-3116. (10.1038/s41418-020-0564-6) 32457483 PMC7560696

[18] Fletcher-Sananikone E et al. 2021 Elimination of radiation-induced senescence in the brain tumor microenvironment attenuates glioblastoma recurrence. Cancer Res. **81**, 5935-5947. (10.1158/0008-5472.CAN-21-0752) 34580063 PMC9724593

[19] Aguayo-Mazzucato C et al. 2019 Acceleration of *β* cell aging determines diabetes and senolysis improves disease outcomes. Cell Metab. **30**, 129-142.e4. (10.1016/j.cmet.2019.05.006) 31155496 PMC6610720

[20] Lee S et al. 2021 Virus-induced senescence is a driver and therapeutic target in COVID-19. Nature **599**, 283-289. (10.1038/s41586-021-03995-1) 34517409

[21] He Y, Li W, Lv D, Zhang X, Zhang X, Ortiz YT, Budamagunta V, Campisi J, Zheng G, Zhou D. 2020 Inhibition of USP7 activity selectively eliminates senescent cells in part via restoration of p53 activity. Aging Cell **19**, 1-11. (10.1111/acel.13117) PMC7059172 32064756

[22] Jeon OH et al. 2017 Local clearance of senescent cells attenuates the development of post-traumatic osteoarthritis and creates a pro-regenerative environment. Nat. Med. **23**, 775-781. (10.1038/nm.4324) 28436958 PMC5785239

[23] Faust HJ et al. 2020 IL-17 and immunologically induced senescence regulate response to injury in osteoarthritis. J. Clin. Invest. **130**, 5493-5507. (10.1172/JCI134091) 32955487 PMC7524483

[24] Chae JB et al. 2021 Targeting senescent retinal pigment epithelial cells facilitates retinal regeneration in mouse models of age-related macular degeneration. GeroScience **43**, 2809-2833. (10.1007/s11357-021-00457-4) 34601706 PMC8602547

[25] Xu M et al. 2018 Senolytics improve physical function and increase lifespan in old age. Nat. Med. **24**, 1246-1256. (10.1038/s41591-018-0092-9) 29988130 PMC6082705

[26] Camell CD et al. 2021 Senolytics reduce coronavirus-related mortality in old mice. Science **373**, eabe4832. (10.1126/science.abe4832) 34103349 PMC8607935

[27] Baar MP et al. 2017 Targeted apoptosis of senescent cells restores tissue homeostasis in response to chemotoxicity and aging. Cell **169**, 132-147.e16. (10.1016/j.cell.2017.02.031) 28340339 PMC5556182

[28] Le HH et al. 2021 Molecular modelling of the FOXO4-TP53 interaction to design senolytic peptides for the elimination of senescent cancer cells. EBioMedicine **73**, 103646. (10.1016/j.ebiom.2021.103646) 34689087 PMC8546421

[29] Meng J et al. 2021 Targeting senescence-like fibroblasts radiosensitizes non-small cell lung cancer and reduces radiation-induced pulmonary fibrosis. JCI Insight **6**, e146334. (10.1172/jci.insight.146334) 34877934 PMC8675198

[30] Fuhrmann-Stroissnigg H et al. 2017 Identification of HSP90 inhibitors as a novel class of senolytics. Nat. Commun. **8**, 1-14. (10.1038/s41467-017-00314-z) 28871086 PMC5583353

[31] Wakita M et al. 2020 A BET family protein degrader provokes senolysis by targeting NHEJ and autophagy in senescent cellsby targeting NHEJ and autophagy in senescent cells. Nat. Commun. **11**, 1-13. (10.1038/s41467-020-15719-6) 32321921 PMC7176673

[32] Go S, Kang M, Kwon SP, Jung M, Jeon OH, Kim BS. 2021 The senolytic drug JQ1 removes senescent cells via ferroptosis. Tissue Eng. Regen. Med. **18**, 841-850. (10.1007/s13770-021-00346-z) 34003467 PMC8440740

[33] Guerrero A et al. 2019 Cardiac glycosides are broad-spectrum senolytics. Nat. Metab. **1**, 1074-1088. (10.1038/s42255-019-0122-z) 31799499 PMC6887543

[34] L'Hôte V, Courbeyrette R, Pinna G, Cintrat JC, Le Pavec G, Delaunay-Moisan A, Mann C, Thuret JY. 2021 Ouabain and chloroquine trigger senolysis of BRAF-V600E-induced senescent cells by targeting autophagy. Aging Cell **20**, 1-14. (10.1111/acel.13447) PMC8564827 34355491

[35] Triana-Martínez F et al. 2019 Identification and characterization of cardiac glycosides as senolytic compounds. Nat. Commun. **10**, 1-12. (10.1038/s41467-019-12888-x) 31636264 PMC6803708

[36] Nogueira-Recalde U et al. 2019 Fibrates as drugs with senolytic and autophagic activity for osteoarthritis therapy. EBioMedicine **45**, 588-605. (10.1016/j.ebiom.2019.06.049) 31285188 PMC6642320

[37] Johmura Y et al. 2021 Senolysis by glutaminolysis inhibition ameliorates various age-associated disorders. Science **371**, 265-270. (10.1126/science.abb5916) 33446552

[38] Wang Y, Chang J, Liu X, Zhang X, Zhang S, Zhang X, Zhou D, Zheng G. 2016 Discovery of piperlongumine as a potential novel lead for the development of senolytic agents. Aging (Albany NY) **8**, 2915-2926. (10.18632/aging.101100) 27913811 PMC5191878

[39] Zhang X et al. 2018 Oxidation resistance 1 is a novel senolytic target. Aging Cell **17**, 1-14. (10.1111/acel.12780) PMC6052462 29766639

[40] Kurz DJ, Decary S, Hong Y, Erusalimsky JD. 2000 Senescence-associated β-galactosidase reflects an increase in lysosomal mass during replicative ageing of human endothelial cells. J. Cell Sci. **113**, 3613-3622. (10.1242/jcs.113.20.3613) 11017877

[41] Hernandez-Segura A, Nehme J, Demaria M. 2018 Hallmarks of cellular senescence. Trends Cell Biol. **28**, 436-453. (10.1016/j.tcb.2018.02.001) 29477613

[42] Glab JA, Mbogo GW, Puthalakath H. 2017 BH3-only proteins in health and disease. Int. Rev. Cell Mol. Biol. **328**, 163-196. 28069133 10.1016/bs.ircmb.2016.08.005

[43] Kale J, Osterlund EJ, Andrews DW. 2018 BCL-2 family proteins: changing partners in the dance towards death. Cell Death Differ. **25**, 65-80. (10.1038/cdd.2017.186) 29149100 PMC5729540

[44] Lomonosova E, Chinnadurai G. 2009 BH3-only proteins in apoptosis and beyond : an overview. Oncogene **27**, 2-19. (10.1038/onc.2009.39) PMC2928556 19641503

[45] Delbridge ARD, Grabow S, Strasser A, Vaux DL. 2016 Thirty years of BCL-2: translating cell death discoveries into novel cancer therapies. Nat. Rev. Cancer **16**, 99-109. (10.1038/nrc.2015.17) 26822577

[46] Timucin AC, Basaga H, Kutuk O. 2019 Selective targeting of antiapoptotic BCL-2 proteins in cancer. Med. Res. Rev. **39**, 146-175. (10.1002/med.21516) 29846950

[47] Gayle SS, Sahni JM, Webb BM, Weber-Bonk KL, Shively MS, Spina R, Bar EE, Summers MK, Keri RA. 2019 Targeting BCL-xL improves the efficacy of bromodomain and extra-terminal protein inhibitors in triple-negative breast cancer by eliciting the death of senescent cells. J. Biol. Chem. **294**, 875-886. (10.1074/jbc.RA118.004712) 30482844 PMC6341404

[48] Rincheval V, Renaud F, Lemaire C, Godefroy N, Trotot P, Boulo V, Mignotte B, Vayssière JL. 2002 Bcl-2 can promote p53-dependent senescence versus apoptosis without affecting the G1/S transition. Biochem. Biophys. Res. Commun. **298**, 282-288. (10.1016/S0006-291X(02)02454-3) 12387829

[49] Kracikova M, Akiri G, George A, Sachidanandam R, Aaronson SA. 2013 A threshold mechanism mediates p53 cell fate decision between growth arrest and apoptosis. Cell Death Differ. **20**, 576-588. (10.1038/cdd.2012.155) 23306555 PMC3595483

[50] Dikovskaya D et al. 2015 Mitotic stress is an integral part of the oncogene-induced senescence program that promotes multinucleation and cell cycle arrest. Cell Rep. **12**, 1483-1496. (10.1016/j.celrep.2015.07.055) 26299965 PMC4562906

[51] Yosef R et al. 2016 Directed elimination of senescent cells through inhibition of Bcl-w and Bcl-xl. Nat. Commun. **7**, 1-11. (10.1038/ncomms11190) PMC4823827 27048913

[52] Zhu Y et al. 2016 Identification of a novel senolytic agent, navitoclax, targeting the Bcl-2 family of anti-apoptotic factors. Aging Cell **15**, 428-435. (10.1111/acel.12445) 26711051 PMC4854923

[53] Chong SJF, Marchi S, Petroni G, Kroemer G, Galluzzi L, Pervaiz S. 2020 Noncanonical cell fate regulation by Bcl-2 proteins. Trends Cell Biol. **30**, 537-555. (10.1016/j.tcb.2020.03.004) 32307222

[54] Chang J et al. 2016 Clearance of senescent cells by ABT263 rejuvenates aged hematopoietic stem cells in mice. Nat. Med. **22**, 78-83. (10.1038/nm.4010) 26657143 PMC4762215

[55] Saleh T et al. 2020 Clearance of therapy-induced senescent tumor cells by the senolytic ABT-263 via interference with BCL-XL–BAX interaction. Mol. Oncol. **14**, 2504-2519. (10.1002/1878-0261.12761) 32652830 PMC7530780

[56] Yamamoto M, Kitanaka C. 2021 ET-6 Gemcitabine radiosensitization primes irradiated malignant meningioma cells for senolytic elimination by navitoclax. Neuro-Oncol. Adv. **3**(Supplement_6), vi4-vi5. (10.1093/noajnl/vdab159.016) PMC8577526 34765973

[57] Malaquin N, Vancayseele A, Gilbert S, Antenor-Habazac L, Olivier MA, Ait Ali Brahem Z, Saad F, Delouya G, Rodier F. 2020 DNA damage- but not enzalutamide-induced. Cells **9**, 1593. (10.3390/cells9071593) 32630281 PMC7408442

[58] Zhan Q, Bieszczad CK, Bae I, Fornace AJ, Craig RW. 1997 Induction of BCL2 family member MCL1 as an early response to DNA damage. Oncogene **14**, 1031-1039. (10.1038/sj.onc.1200927) 9070651

[59] Miyashita T, Krajewski S, Krajewska M, Wang HG, Lin HK, Liebermann DA, Hoffman B, Reed JC. 1994 Tumor suppressor p53 is a regulator of bcl-2 and bax gene expression in vitro and in vivo. Oncogene **9**, 1799-1805. 8183579

[60] Widden H, Placzek WJ. 2021 The multiple mechanisms of MCL1 in.the regulation of cell fate. Commun. Biol. **4**, 1-12. (10.1038/s42003-021-02564-6) 34475520 PMC8413315

[61] Jochems F et al. 2021 The cancer SENESCopedia: a delineation of cancer cell senescence. Cell Rep. **36**, 109441. (10.1016/j.celrep.2021.109441) 34320349 PMC8333195

[62] Schwarzenbach C, Tatsch L, Vilar JB, Rasenberger B, Beltzig L, Kaina B, Tomicic MT, Christmann M. 2021 Targeting c-iap1, c-iap2, and bcl-2 eliminates senescent glioblastoma cells following temozolomide treatment. Cancers (Basel) **13**, 3585. (10.3390/cancers13143585) 34298797 PMC8306656

[63] Rahman M et al. 2022 Selective vulnerability of senescent glioblastoma cells to Bcl-XL inhibition. Mol. Cancer Res. **20**, 938-948. (10.1101/2020.06.03.132712) 35191501 PMC9196639

[64] Zhu Y et al. 2017 New agents that target senescent cells : the flavone, fisetin, and the BCL - X L inhibitors, A1331852 and A1155463. Aging (Albany NY) **9**, 955-963. (10.18632/aging.101202) 28273655 PMC5391241

[65] Hartman ML, Czyz M. 2020 BCL-w: apoptotic and non-apoptotic role in health and disease. Cell Death Dis. **11**, 1-16. (10.1038/s41419-020-2417-0) 32317622 PMC7174325

[66] Tchkonia T, Morbeck DE, Von Zglinicki T, Van Deursen J, Lustgarten J, Scrable H, Khosla S, Jensen MD, Kirkland JL. 2010 Fat tissue, aging, and cellular senescence. Aging Cell **9**, 667-684. (10.1111/j.1474-9726.2010.00608.x) 20701600 PMC2941545

[67] Edlich F. 2018 BCL-2 proteins and apoptosis: recent insights and unknowns. Biochem. Biophys. Res. Commun. **500**, 26-34. (10.1016/j.bbrc.2017.06.190) 28676391

[68] Lafontaine J, Cardin GB, Malaquin N, Boisvert JS, Rodier F, Wong P. 2021 Senolytic targeting of bcl-2 anti-apoptotic family increases cell death in irradiated sarcoma cells. Cancers (Basel) **13**, 1-20. (10.3390/cancers13030386) PMC7866159 33494434

[69] Del Gaizo Moore V, Letai A. 2013 BH3 profiling – Measuring integrated function of the mitochondrial apoptotic pathway to predict cell fate decisions. Cancer Lett. **332**, 202-205. (10.1016/j.canlet.2011.12.021) 22230093 PMC3770266

[70] Montero J et al. 2015 Drug-Induced death signaling strategy rapidly predicts cancer response to chemotherapy. Cell **160**, 977-989. (10.1016/j.cell.2015.01.042) 25723171 PMC4391197

[71] Montero J, Letai A. 2018 Why do BCL-2 inhibitors work and where should we use them in the clinic? Cell Death Differ. **25**, 56-64. (10.1038/cdd.2017.183) 29077093 PMC5729538

[72] Potter DS, Du R, Bhola P, Bueno R, Letai A. 2021 Dynamic BH3 profiling identifies active BH3 mimetic combinations in non-small cell lung cancer. Cell Death Dis. **12**, 1-12. (10.1038/s41419-021-04029-4) 34315868 PMC8316436

[73] Oltersdorf T et al. 2005 An inhibitor of Bcl-2 family proteins induces regression of solid tumours. Nature **435**, 677-681. (10.1038/nature03579) 15902208

[74] Wilson WH et al. 2010 Navitoclax, a targeted high-affinity inhibitor of BCL-2, in lymphoid malignancies: a phase 1 dose-escalation study of safety, pharmacokinetics, pharmacodynamics, and antitumour activity. Lancet Oncol. **11**, 1149-1159. (10.1016/S1470-2045(10)70261-8) 21094089 PMC3025495

[75] González-Gualda E et al. 2020 Galacto-conjugation of Navitoclax as an efficient strategy to increase senolytic specificity and reduce platelet toxicity. Aging Cell **19**, e13142. (10.1111/acel.13142) 32233024 PMC7189993

[76] He Y et al. 2020 Using proteolysis-targeting chimera technology to reduce navitoclax platelet toxicity and improve its senolytic activity. Nat. Commun. **11**, 1-14. (10.1038/s41467-020-15838-0) 32332723 PMC7181703

[77] Galiana I, Lozano-Torres B, Sancho M, Alfonso M, Bernardos A, Bisbal V, Serrano M, Martínez-Máñez R, Orzaez M. 2020 Preclinical antitumor efficacy of senescence-inducing chemotherapy combined with a nanoSenolytic. J. Control Release **323**, 624-634. (10.1016/j.jconrel.2020.04.045) 32376460

[78] Negi A, Voisin-Chiret AS. 2022 Strategies to reduce the on-target platelet toxicity of Bcl-xL inhibitors: PROTACs, SNIPERs and Prodrug-based approaches. ChemBioChem **23**, 202100689. (10.1002/cbic.202100689) PMC9311450 35263486

[79] Wajapeyee N, Serra RW, Zhu X, Mahalingam M, Green MR. 2008 Oncogenic BRAF induces senescence and apoptosis through pathways mediated by the secreted protein IGFBP7. Cell **132**, 363-374. (10.1016/j.cell.2007.12.032) 18267069 PMC2266096

[80] Coppé JP, Desprez PY, Krtolica A, Campisi J. 2010 The senescence-associated secretory phenotype: the dark side of tumor suppression. Annu. Rev. Pathol. Mech. Dis. **5**, 99-118. (10.1146/annurev-pathol-121808-102144) PMC4166495 20078217

[81] Beauséjour CM, Krtolica A, Galimi F, Narita M, Lowe SW, Yaswen P, Campisi J. 2003 Reversal of human cellular senescence: roles of the p53 and p16 pathways. EMBO J. **22**, 4212-4222. (10.1093/emboj/cdg417) 12912919 PMC175806

[82] Johmura Y et al. 2016 SCF Fbxo22-KDM4A targets methylated p53 for degradation and regulates senescence. Nat. Commun. **7**, 1-12. (10.1038/ncomms10574) PMC4754341 26868148

[83] Herranz N, Gil J, Herranz N, Gil J. 2018 Mechanisms and functions of cellular senescence. J. Clin. Invest. **128**, 1238-1246. (10.1172/JCI95148) 29608137 PMC5873888

[84] Sheekey E, Narita M. 2022 p53 in senescence—it's a marathon not a sprint. FEBS J. (10.1111/febs.16325) 34921507

[85] Yosef R et al. 2017 p21 maintains senescent cell viability under persistent DNA damage response by restraining JNK and caspase signaling. EMBO J. **36**, 2280-2295. (10.15252/embj.201695553) 28607003 PMC5538795

[86] Dankort D et al. 2009 BrafV600E cooperates with Pten loss to induce metastatic melanoma. Nat. Genet. **41**, 544-552. (10.1038/ng.356) 19282848 PMC2705918

[87] De Keizer PLJ et al. 2010 Activation of forkhead box O transcription factors by oncogenic BRAF promotes p21cip1-dependent senescence. Cancer Res. **70**, 8526-8536. (10.1158/0008-5472.CAN-10-1563) 20959475 PMC2989643

[88] Bourgeois B, Madl T. 2018 Regulation of cellular senescence via the FOXO4-p53 axis. FEBS Lett. **592**, 2083-2097. (10.1002/1873-3468.13057) 29683489 PMC6033032

[89] Zhang C et al. 2020 FOXO4-DRI alleviates age-related testosterone secretion insufficiency by targeting senescent Leydig cells in aged mice. Aging (Albany NY **12**, 1272-1284. (10.18632/aging.102682) 31959736 PMC7053614

[90] Huang Y, He Y, Makarcyzk MJ, Lin H. 2021 Senolytic peptide FOXO4-DRI selectively removes senescent cells from in vitro expanded human chondrocytes. Front. Bioeng. Biotechnol. **9**, 1-9. 10.3389/fbioe.2021.677576 PMC8116695 33996787

[91] Lau JL, Dunn MK. 2018 Therapeutic peptides: historical perspectives, current development trends, and future directions. Bioorganic Med. Chem. **26**, 2700-2707. (10.1016/j.bmc.2017.06.052) 28720325

[92] Kwon SK, Saindane M, Baek KH. 2017 P53 stability is regulated by diverse deubiquitinating enzymes. Biochim. Biophys. Acta Rev. Cancer **1868**, 404-411. (10.1016/j.bbcan.2017.08.001) 28801249

[93] Dolgin E. 2020 Send in the senolytics. Nat. Biotechnol. **38**, 1371-1377. (10.1038/s41587-020-00750-1) 33184478

[94] Sánchez-Rivera FJ et al. 2021 Mitochondrial apoptotic priming is a key determinant of cell fate upon p53 restoration. Proc. Natl Acad. Sci. USA **118**, 1-8. (10.1073/pnas.2019740118) PMC8201929 34074758

[95] Osaki M, Oshimura M, Ito H. 2004 PI3 K-Akt pathway: its functions and alterations in human cancer. Apoptosis **9**, 667-676. (10.1023/B:APPT.0000045801.15585.dd) 15505410

[96] Manning BD, Toker A. 2017 AKT/PKB signaling: navigating the network. Cell **169**, 381-405. (10.1016/j.cell.2017.04.001) 28431241 PMC5546324

[97] Karaman MW et al. 2008 A quantitative analysis of kinase inhibitor selectivity. Nat. Biotechnol. **26**, 127-132. (10.1038/nbt1358) 18183025

[98] Li J et al. 2010 A chemical and phosphoproteomic characterization of dasatinib action in lung cancer. Nat. Chem. Biol. **6**, 291-299. (10.1038/nchembio.332) 20190765 PMC2842457

[99] Hsieh HH, Chen YC, Jhan JR, Lin JJ. 2017 The serine protease inhibitor serpinB2 binds and stabilizes p21 in.senescent cells. J. Cell Sci. **130**, 3272-3281. 28794016 10.1242/jcs.204974

[100] Tonnetti L, Netzel-Arnett S, Darnell GA, Hayes T, Buzza MS, Anglin IE, Suhrbier A, Antalis TM. 2008 SerpinB2 protection of retinoblastoma protein from calpain enhances tumor cell survival. Cancer Res. **68**, 5648-5657. (10.1158/0008-5472.CAN-07-5850) 18632617 PMC2561898

[101] Anerillas C et al. 2022 Early SRC activation skews cell fate from apoptosis to senescence. Sci. Adv. **8**, 1-17. (10.1126/sciadv.abm0756) PMC8993123 35394839

[102] Pungsrinont T, Sutter MF, Ertingshausen MCCM, Lakshmana G, Kokal M, Khan AS, Baniahmad A. 2020 Senolytic compounds control a distinct fate of androgen receptor agonist-and antagonist-induced cellular senescent LNCaP prostate cancer cells. Cell Biosci. **10**, 1-13. (10.1186/s13578-020-00422-2) 32351687 PMC7183592

[103] Courtois-Cox S, Jones SL, Cichowski K. 2008 Many roads lead to oncogene-induced senescence. Oncogene **27**, 2801-2809. (10.1038/sj.onc.1210950) 18193093

[104] Wang C et al. 2019 Inducing and exploiting vulnerabilities for the treatment of liver cancer. Nature **574**, 268-272. (10.1038/s41586-019-1607-3) 31578521 PMC6858884

[105] Halkoum R, Salnot V, Capallere C, Plaza C, L'honoré A, Pays K, Friguet B, Nizard C, Petropoulos I. 2022 Glyoxal induces senescence in human keratinocytes through oxidative stress and activation of the protein kinase B/FOXO3a/p27KIP1 pathway. J. Invest. Dermatol. **142**, 2068-2078.e7. (10.1016/j.jid.2021.12.022) 34971698

[106] Ishikawa S, Ishikawa F. 2020 Proteostasis failure and cellular senescence in long-term cultured postmitotic rat neurons. Aging Cell **19**, 1-15. (10.1111/acel.13071) PMC6974705 31762159

[107] Sabath N, Levy-Adam F, Younis A, Rozales K, Meller A, Hadar S, Soueid-Baumgarten S, Shalgi R. 2020 Cellular proteostasis decline in human senescence. Proc. Natl Acad. Sci. USA **117**, 31 902-31 913. (10.1073/pnas.2018138117) PMC7749315 33257563

[108] Ward CP et al. 2022 Aging alters the metabolic flux signature of the ER-unfolded protein response in vivo in mice. Aging Cell **21**, 1-13. (10.1111/acel.13558) PMC8920450 35170180

[109] Abbadie C, Pluquet O. 2020 Unfolded protein response (UPR) controls major senescence hallmarks. Trends Biochem. Sci. **45**, 371-374. (10.1016/j.tibs.2020.02.005) 32311331

[110] Martinez-Miguel VE et al. 2021 Increased fidelity of protein synthesis extends lifespan. Cell Metab. **33**, 2288-2300.e12. (10.1016/j.cmet.2021.08.017) 34525330 PMC8570412

[111] Yamamoto-Imoto H et al. 2022 Age-associated decline of MondoA drives cellular senescence through impaired autophagy and mitochondrial homeostasis. Cell Rep. **38**, 110444. (10.1016/j.celrep.2022.110444) 35235784

[112] Bialik S, Dasari SK, Kimchi A. 2018 Autophagy-dependent cell death - where, how and why a cell eats itself to death. J. Cell Sci. **131**, jcs215152. (10.1242/jcs.215152) 30237248

[113] Fujii S et al. 2012 Insufficient autophagy promotes bronchial epithelial cell senescence in chronic obstructive pulmonary disease. Oncoimmunology **1**, 630-641. (10.4161/onci.20297) 22934255 PMC3429567

[114] Gammoh N et al. 2016 Suppression of autophagy impedes glioblastoma development and induces senescence. Autophagy **12**, 1431-1439. (10.1080/15548627.2016.1190053) 27304681 PMC5082770

[115] Schepers A, Jochems F, Lieftink C, Wang L, Pogacar Z, de Oliveira RL, De Conti G, Beijersbergen RL, Bernards R. 2021 Identification of autophagy-related genes as targets for senescence induction using a customizable CRISPR-based suicide switch screen. Mol. Cancer Res. **19**, 1613-1621. (10.1158/1541-7786.MCR-21-0146) 34158393 PMC7611779

[116] Gewirtz DA. 2013 Autophagy and senescence: a partnership in search of definition. Autophagy **9**, 808-812. (10.4161/auto.23922) 23422284 PMC3669198

[117] Liu H, He Z, Von Rütte T, Yousefi S, Hunger RE, Simon HU. 2013 Down-regulation of autophagy-related protein 5 (ATG5) contributes to the pathogenesis of early-stage cutaneous melanoma. Sci. Transl. Med. **5**, 202ra123. (10.1126/scitranslmed.3005864) 24027027

[118] Kwon Y, Kim JW, Jeoung JA, Kim MS, Kang C. 2017 Autophagy is pro-senescence when seen in close-up, but anti-senescence in long-shot. Mol. Cells **40**, 607-612. (10.14348/molcells.2017.2279) 28927262 PMC5638768

[119] Lee Y et al. 2021 Coordinate regulation of the senescent state by selective autophagy. Dev. Cell **56**, 1512-1525.e7. (10.1016/j.devcel.2021.04.008) 33915088

[120] Masaldan S et al. 2018 Iron accumulation in senescent cells is coupled with impaired ferritinophagy and inhibition of ferroptosis. Redox Biol. **14**(September 2017), 100-115. (10.1016/j.redox.2017.08.015) 28888202 PMC5596264

[121] Del Rey MJ et al. 2019 Senescent synovial fibroblasts accumulate prematurely in rheumatoid arthritis tissues and display an enhanced inflammatory phenotype. Immun. Ageing **16**, 1-9. (10.1186/s12979-019-0169-4) 31708994 PMC6833299

[122] Courtois-Cox S, Genther Williams SM, Reczek EE, Johnson BW, McGillicuddy LT, Johannessen CM, Hollstein PE, Maccollin M, Cichowski K. 2006 A negative feedback signaling network underlies oncogene-induced senescence. Cancer Cell **10**, 459-472. (10.1016/j.ccr.2006.10.003) 17157787 PMC2692661

[123] Kennedy AL et al. 2011 Activation of the PIK3CA/AKT pathway suppresses senescence induced by an activated RAS oncogene to promote tumorigenesis. Mol. Cell **42**, 36-49. (10.1016/j.molcel.2011.02.020) 21474066 PMC3145340

[124] Deryabin PI, Shatrova AN, Borodkina AV. 2021 Apoptosis resistance of senescent cells is an intrinsic barrier for senolysis induced by cardiac glycosides. Cell Mol. Life Sci. **78**, 7757-7776. (10.1007/s00018-021-03980-x) 34714358 PMC8629786

[125] Moiseeva O, Bourdeau V, Roux A, Deschênes-Simard X, Ferbeyre G. 2009 Mitochondrial dysfunction contributes to oncogene-induced senescence. Mol. Cell Biol. **29**, 4495-4507. (10.1128/MCB.01868-08) 19528227 PMC2725737

[126] Wu M et al. 2017 Metabolomics-proteomics combined approach identifies differential metabolism-associated molecular events between senescence and apoptosis. J. Proteome Res. **16**, 2250-2261. (10.1021/acs.jproteome.7b00111) 28467092

[127] Ziegler DV, Martin N, Bernard D. 2021 Cellular senescence links mitochondria-ER contacts and aging. Commun. Biol. **4**, 1-14. (10.1038/s42003-021-02840-5) 34819602 PMC8613202

[128] Farfariello V et al. 2022 TRPC3 shapes the ER-mitochondria Ca2+ transfer characterizing tumour-promoting senescence. Nat. Commun. **13**, 1-18. (10.1038/s41467-022-28597-x) 35177596 PMC8854551

[129] Zhong G, Qin S, Townsend D, Schulte BA, Tew KD, Wang GY. 2019 Oxidative stress induces senescence in breast cancer stem cells. Biochem. Biophys. Res. Commun. **514**, 1204-1209. (10.1016/j.bbrc.2019.05.098) 31109646 PMC6556123

[130] Ngoi NY, Liew AQ, Chong SJF, Davids MS, Clement MV, Pervaiz S. 2021 The redox-senescence axis and its therapeutic targeting. Redox Biol. **45**, 102032. (10.1016/j.redox.2021.102032) 34147844 PMC8220395

[131] Yang D, Tian X, Ye Y, Liang Y, Zhao J, Wu T, Lu N. 2021 Identification of GL-V9 as a novel senolytic agent against senescent breast cancer cells. Life Sci. **272**, 119196. (10.1016/j.lfs.2021.119196) 33617857

[132] Xu Q et al. 2021 The flavonoid procyanidin C1 has senotherapeutic activity and increases lifespan in mice. Nat. Metab. **3**, 1706-1726. (10.1038/s42255-021-00491-8) 34873338 PMC8688144

[133] Fleury H et al. 2019 Exploiting interconnected synthetic lethal interactions between PARP inhibition and cancer cell reversible senescence. Nat. Commun. **10**, 1-15. (10.1038/s41467-019-10460-1) 31186408 PMC6560032

[134] Lujambio A. 2016 To clear, or not to clear (senescent cells)? That is the question. Insid Cell **1**, 87-95. (10.1002/bies.201670910) 27417123

[135] Grosse L, Wagner N, Emelyanov A, Molina C, Lacas-Gervais S, Wagner KD, Bulavin DV. 2020 Defined p16 high senescent cell types are indispensable for mouse healthspan. Cell Metab. **32**, 87-99.e6. (10.1016/j.cmet.2020.05.002) 32485135

[136] Martin N, Huna A, Bernard D. 2021 Elimination of senescent endothelial cells: good or bad idea? Trends Cell Biol. **31**, 327-330. (10.1016/j.tcb.2021.02.009) 33715897

[137] Cai Y et al. 2020 Elimination of senescent cells by *β* -galactosidase-targeted prodrug attenuates inflammation and restores physical function in aged mice. Cell Res. **30**, 574-589. (10.1038/s41422-020-0314-9) 32341413 PMC7184167

[138] Muñoz-Espín D et al. 2018 A versatile drug delivery system targeting senescent cells. EMBO Mol. Med. **10**, 1-18. (10.15252/emmm.201708365) 30012580 PMC6127887

[139] Li W, He Y, Zhang R, Zheng G, Zhou D. 2019 The curcumin analog EF24 is a novel senolytic agent. Aging (Albany NY) **11**, 771-782. (10.18632/aging.101787) 30694217 PMC6366974

[140] Nelson KM, Dahlin JL, Bisson J, Graham J, Pauli GF, Walters MA. 2017 The essential medicinal chemistry of curcumin. J. Med. Chem. **60**, 1620-1637. (10.1021/acs.jmedchem.6b00975) 28074653 PMC5346970

[141] Heger M. 2017 Don't discount all curcumin trial data. Nature **543**, 40. (10.1038/543040c) PMC6814206 28252078

[142] Cherif H, Bisson D, Jarzem P, Weber M, Ouellet J, Haglund L. 2019 Curcumin and o-Vanillin exhibit evidence of senolytic activity in human IVD cells in vitro. J. Clin. Med. **8**, 433. (10.3390/jcm8040433) 30934902 PMC6518239

[143] Cherif H, Bisson DG, Mannarino M, Rabau O, Ouellet JA, Haglund L. 2020 Senotherapeutic drugs for human intervertebral disc degeneration and low back pain. Elife **9**, 1-25. (10.7554/eLife.54693) PMC7442487 32821059

[144] Yousefzadeh MJ et al. 2018 Fisetin is a senotherapeutic that extends health and lifespan. EBioMedicine **36**, 18-28. (10.1016/j.ebiom.2018.09.015) 30279143 PMC6197652

[145] Wiley CD et al. 2021 Oxylipin biosynthesis reinforces cellular senescence and allows detection of senolysis. Cell Metab. **33**, 1124-1136.e5. (10.1016/j.cmet.2021.03.008) 33811820 PMC8501892

